# *Malassezia* Yeasts: How Many Species Infect Humans and Animals?

**DOI:** 10.1371/journal.ppat.1003892

**Published:** 2014-02-27

**Authors:** F. Javier Cabañes

**Affiliations:** Veterinary Mycology Group, Department of Animal Health and Anatomy, Veterinary School, Universitat Autònoma de Barcelona, Bellaterra, Catalonia, Spain; Duke University Medical Center, United States of America

## The Main Significance of *Malassezia* Yeasts and Their Position in the Tree of Life

*Malassezia* species are lipophilic yeasts that are members of the normal mycobiota of the skin and mucosal sites of a variety of homeothermic animals. They are also among the few basidiomycetous fungi, such as some *Cryptococcus* spp., *Rhodotorula* spp., and *Trichosporon* spp., that can produce disease in man and animals. However, in contrast with these other species, which are quite often involved in disseminated infections in immunosuppressed patients, *Malassezia* yeasts are associated mainly with certain skin diseases [1].

This special lipophilic group of yeasts is unique among the fungi. Phylogenetically, they form a well-defined cluster of skin-living yeasts, surrounded by plant pathogens and phylloplane-inhabiting fungi (e.g., *Ustilago, Tilletiopsis*). However, the taxonomic position of the genus *Malassezia* in the classes of the phylum Basidiomycota is not yet totally well defined. Moreover, the sexual form of these yeasts is still unknown. Recently, a region corresponding to the mating type locus (MAT) has been identified for these yeasts, and it has been suggested that if there is an extant sexual cycle for some of these yeasts that it is more likely to be bipolar, with just two mating types, rather than tetrapolar, with many mating types [2].

In the last higher-level fungal phylogenetic classification revision [3], the monophyletic genus *Malassezia* was the only genus included in the order Malasseziales, which has an uncertain taxonomic position in the subphylum Ustilagomycotina (e.g., smut fungi). Very recently, the class Malasseziomycetes has been proposed to accommodate these fungi (2013, provided from an anonymous reviewer; unreferenced). They are taxonomically distant to the orders which include the other commented pathogenic basidiomycetous yeasts of the genera *Cryptococcus* (Filobasidiales) and *Trichosporon* in Agaricomycotina (e.g., mushrooms) and of the polyphyletic genus *Rhodotorula* (Sporidiobolales and Cystobasidiales) in Pucciniomycotina (e.g., rust fungi).

## Spectrum of *Malassezia* Species That Infect Humans and Animals

At present, the genus *Malassezia* includes 14 species ([4]; Table 1), all of which infect or colonize humans or animals. However, until the late 1980s, this genus remained limited to only to two species; one of these, *M. furfur* (sensu lato), was considered a heterogeneous group of lipid-dependent yeasts living on human skin and requiring long-chain fatty acids to grow, while the lipophilic but non–lipid-dependent species *M. pachydermatis* was restricted to animal skin. The latter is the only species in the genus that does not require lipid supplementation for development in culture medium. *M. sympodialis*, a lipid-dependent species isolated from human skin, was the third species accepted in the genus, a century after the description of *M. furfur* [5]. Later, the genus *Malassezia* was revised on the basis of morphological, physiological, and rRNA gene sequencing studies, and four new lipid-dependent species were described [6]. At the same time, different studies [7]–[9] confirmed that the skin of healthy animals could also be colonized by lipid-dependent species, in addition to the non–lipid-dependent species *M. pachydermatis*. These lipid-dependent species are the major component of the lipophilic mycobiota occurring on the skin of horses and various ruminants [10]. Some of these yeasts isolated from animals were described subsequently as new species, such as *M. nana* [11], *M. equina*, or *M. caprae* [12]. Nowadays, *Malassezia* yeasts have been isolated from almost all domestic animals, different wild animals held in captivity, and also from wildlife [1]. Despite this, the occurrence of *Malassezia* yeasts on the skin of most animals remains unknown. The observed host specificity of some of these species made it possible to anticipate an increase in the number of new species in this genus, particularly if other animal species, mainly wild species, were studied.

**Table 1:** Current described *Malassezia* species, authorities, year of the description, and their main hosts^a^ [24].

*Malassezia* species	Main host/others
*M. furfur*, (Robin) Baillon, 1889	Man/cow, elephant, pig, monkey, ostrich, pelican
*M. pachydermatis*, (Weidman) Dodge, 1925	Dog, cat/carnivores, birds
*M. sympodialis*, Simmons & Guého, 1990	Man/horse, pig, sheep
*M. globosa*, Midgley et al., 1996	Man/cheetah, cow
*M. obtusa*, Midgley et al., 1996	Man
*M. restricta*, Guého et al., 1996	Man
*M. slooffiae*, Guillot et al., 1996	Man, pig/goat, sheep
*M. dermatis*, Sugita et al., 2002	Man
*M. japonica*, Sugita et al., 2003	Man
*M. nana*, Hirai et al., 2004	Cat, cow/dog
*M. yamatoensis*, Sugita et al., 2004	Man
*M. caprae*, Cabañes & Boekhout, 2007	Goat/horse
*M. equina*, Cabañes & Boekhout, 2007	Horse/cow
*M. cuniculi*, Cabañes & Castellá, 2011	Rabbit

a Cited only those species confirmed by rDNA sequencing analysis.

## *Malassezia* Yeasts and Disease

The pathogenic role of *Malassezia* yeasts in skin diseases has always been a matter of controversy. Commensal *Malassezia* yeasts are clearly implicated in human skin diseases without the presence of inflammation but with heavy fungal load, such as pityriasis versicolor. They are also associated with other skin disorders with characteristic inflammation, such as seborrheic dermatitis, atopic dermatitis, folliculitis, and psoriasis, where their role in the pathogenesis is less clear and, in some cases, speculative [13]. Emerging evidence demonstrates that the interaction of *Malassezia* yeasts with the skin is multifaceted and entails constituents of the fungal wall, enzymes, and metabolic products, as well as the cellular components of the epidermis. Some skin disorders can be exacerbated by the interactions between *Malassezia* yeasts and the host immune system [2].

Although *M. globosa* was initially reported to be the main species associated with pityriasis versicolor, subsequent studies have shown that the distribution of *Malassezia* species from healthy and diseased skin is equivalent, thus failing to substantiate the existence of a pathogenic species not only in pityriasis versicolor but also for the other *Malassezia*-associated diseases. *M. globosa* and *M. restricta* are the most commonly found species on healthy and diseased human skin [14]. However, other species such as *M. sympodialis* or *M. furfur* have been also associated with various human skin disorders [15].

On the other hand, mainly *M. furfur* and *M. pachydermatis* have been reported to be the cause of a low percentage of yeast systemic infections. However, fungemia produced by these yeasts may be underdiagnosed by modern automated blood systems for fungal detection if culture media with lipids are not included in the diagnostic protocol [16]. The majority of published case reports and miniepidemics have involved infants, children, and adults with profound immunosuppression, serious concurrent health problems, and the infusion of total parenteral nutrition with lipid supplementation through central vascular catheters. The main ingredients of this nutrition system (i.e., linoleic, oleic, and palmitic acids) are potent growth stimulants for *Malassezia* species [15].

Skin colonization by *Malassezia* species of healthy human neonates does not include *M. pachydermatis*, whereas the occurrence of other species such as *M. sympodialis* and *M. globosa* begins at birth and increases in the first weeks of life. [17]. This fact corroborates the animal origin of *M. pachydermatis* in human infections. Furthermore, it should be noted that zoonotic transfer of *M. pachydermatis* has been documented from dogs to neonates by healthcare workers who own dogs [18].

**Figure 1:**
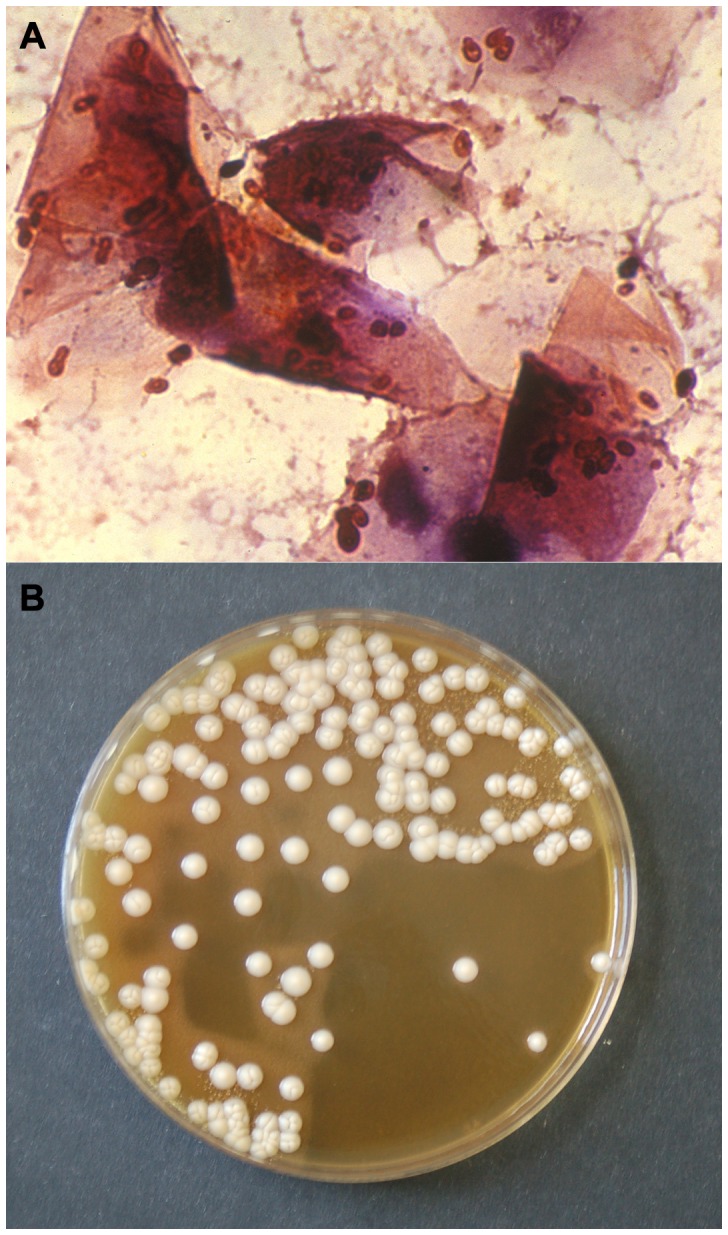
Gram stain of a smear (A) and culture (B) from an otic swab of a dog with otitis externa, showing numerous *M. pachydermatis* cells (A) and colonies (B). This species is a controversial pathogen that is now recognized as an important cause of dermatitis and otitis externa in dogs. Author: F. Javier Cabañes.

*M. pachydermatis*, the only species in the genus that does not require lipid supplementation for development in culture medium, is considered to be zoophilic, and is frequently found on wild and domestic carnivores. This species is usually associated with otitis externa and different kinds of dermatitis in domestic animals, especially in dogs (Figure 1). This species is more frequently isolated from dogs than cats and appears to be a relatively infrequent pathogen in other animals. This yeast seems to have an opportunistic nature, and it may become pathogenic with any detected alteration in the skin surface microclimate or in the host defense. In some canine breeds, hypersensitivity conditions such as flea allergy dermatitis, food hypersensitivity or atopy, and antimicrobial or corticosteroid therapy may be factors favoring proliferation of these yeasts. Lipid-dependent species seem to be found more frequently in cats than in dogs, but very little is known about their pathogenic role in animal skin [19].

## Difficulties in Determining the Biodiversity and Significance of *Malassezia* Yeasts

The study of some *Malassezia* yeasts continues to be difficult, due mainly to their low viability and lack of suitable methods for their isolation and preservation. The majority of yeasts can be stored at temperatures between 4 and 12°C and subcultured at intervals of 6 to 8 months. However, *Malassezia* spp. do not fit this pattern. Freezing at −80°C is the only successful method to maintain viable all *Malassezia* spp., particularly *M. globosa*, *M. restricta*, and *M. obtusa*, which have been reported as difficult species to maintain in vitro [20].

In most surveys, these yeasts have been identified only on the basis of phenotypic characteristics without confirmation through molecular analysis. Difficulties remain in obtaining a high level of certainty in the identification of some lipid-dependent strains by means of physiological tests (e.g., Tween physiological tests) without molecular characterization. Although some *Malassezia* yeasts may be distinguished using these tests, sequencing of some genes (e.g., ITS-5.8S and D1D2 26S rRNA, β-tubulin) [4] or the use of new tools such as MALDI-TOF mass spectrometry [21] are necessary for a proper identification.

Recently, the spectrum of fungal species in the human skin has been explored using culture and culture-independent methods [22]. In this study, *Malassezia* yeasts predominated on most of the sampled body sites. Moreover, 11 of the 14 species (all of them, with the exception of *M. caprae*, *M. cuniculi*, and *M. equina*) (2013 letter from K. Findley to me; unreferenced) were directly identified by rRNA gene sequencing from different clinical samples of ten healthy volunteers. Other DNA sequences that may represent unidentified *Malassezia* spp. were also detected. Some species predominated in certain body sites (e.g., *M. globosa* on the back). Using culturing methods, apparently the most abundant *Malassezia* species on human skin (e.g., *M. globosa*, *M. restricta*, and *M. sympodialis*) were isolated in this study. These authors [22] used only Sabouraud glucose agar (SGA) with olive oil containing chloramphenicol and cycloheximide for recovering *Malassezia* species from different body sites.

The use of SGA overlaid with olive oil has been used frequently in the past, but only some *Malassezia* species grow well on this medium [23], [24]. For an exhaustive survey, the samples must be inoculated onto more complex culture media, such as modified Dixon agar (mDA) or Leeming and Notman agar (LNA), which facilitate the recovery of the more fastidious *Malassezia* species from the skin. These culture media include, among other ingredients, a mixture of fatty acids such as oleic acid, whole-fat cow milk, and some polyoxyethylene sorbitanesters (e.g., Tween 40, Tween 60). However, the exact nutritional requirements of *Malassezia* species in culture are yet to be fully determined, and this hinders the study of these yeasts. Moreover, it is difficult and expensive to obtain fatty acids of sufficient purity to fully establish the fatty-acid requirements of each *Malassezia* species [25].

A recent example of the difficulties inherent to the recovery of these fastidious yeasts from the skin is *M. cuniculi*. In the description of this species [26], only a few lipid- dependent *Malassezia* yeasts were recovered from two of the 11 rabbits investigated. They grew scarcely on LNA and no growth was obtained either on mDA or SGA. They were also not able to grow on glucose peptone agar supplemented with Tweens (20, 40, 60, and 80) and Cremophor EL as sole sources of lipids, which are used to phenotypically characterize these species [6], [27]. This inhibition of growth may be related to the toxic effects of these mixtures of fatty acids at higher concentrations. LNA contains, among other components, Tween 60 at a 10-fold lower concentration (0.05%) than that used in the Tween physiological tests [6].

In other recent surveys performed in soil nematodes [28], marine sponges [29], and coral colonies [30], *Malassezia* yeasts have been tentatively identified exclusively on the basis of some genotypic characteristics by culture-independent methods. However, although other habitats for *Malassezia* yeasts may exist, their significance and the real identity of these yeasts still remain unknown.
